# Quality evaluation of *Arnebia euchroma* with different growth years based on metabolomics and antioxidant activity

**DOI:** 10.3389/fpls.2026.1735489

**Published:** 2026-01-29

**Authors:** Zumrat Obul, Yuan-jin Qiu, Ya-qin Zhao, Jun Zhu, Guo-ping Wang, Wen-dan Song, Aybek Rehmetulla, Cong-zhao Fan, Ji-zhao Zhang

**Affiliations:** 1 College of Traditional Chinese Medicine, Xinjiang Medical University, Urumqi, China; 2 Xinjiang Institute of Materia Medica, Xinjiang Key Laboratory of Chinese Materia Medica and Ethnic Materia Medica, Xinjiang Key Laboratory of Traditional Chinese Medicine Resources Research and Development, Urumqi, Xinjiang, China

**Keywords:** antioxidant *in vitro*, *Arnebia euchroma*, growth years, marker compounds, root biomass, untargeted metabolomics

## Abstract

**Introduction:**

*Arnebia euchroma* (Royle) Johnst., a perennial herb in the Boraginaceae family, is valued for its medicinal properties. The harvesting period is crucial for ensuring both the quality and yield of this medicinal material. However, gaps in the knowledge of its optimal harvesting period impede the establishment of standardized cultivation and quality control protocols. This study aims to analyze the quality variations and their dynamic patterns in *A. euchroma* across different growth years, to provide evidence for determining its optimal harvesting time.

**Methods:**

This study used roots from 2- to 7-year-old *A. euchroma* as the research material. Fresh and dry root weights across multiple growth stages were compared, and key marker compounds were quantified using Ultraviolet–visible(UV) spectrophotometry and High Performance Liquid Chromatography(HPLC). At the same time, metabolite accumulation patterns were profiled via Ultra High Performance Liquid Chromatography-Tandem Mass Spectrometry(UHPLC-MS) untargeted metabolomics. We also applied network pharmacology to identify potential bioactive constituents, and their *in vitro* antioxidant activity was evaluated using 2,2’-azino-bis(3-ethylbenzothiazoline-6-sulfonic acid) (ABTS), 2,2-diphenyl-1-picrylhydrazyl (DPPH), and ferric ion reducing antioxidant potential (FRAP) assays.

**Results:**

This study demonstrated that the fresh and dry weights of *A. euchroma* roots increased steadily and consistently with increasing growth years. Total hydroxynaphthoquinone pigments followed a “rise-decline-rise” trend, The content ranged from 1.73% to 4.44%. The concentration of β,β′-dimethylacrylshikonin increased steadily from 0.11% in 2-year-old plants to 0.52% in 7-year-old plants. Untargeted metabolomic analysis identified 1,058 metabolites, including 355 differentially accumulated metabolites (DAMs). K-means clustering of DAMs revealed distinct accumulation patterns: flavonoids, phenolic acids, polyketides, phenylpropanoids, and fatty acids peaked in 4-year-old plants. Network pharmacology analysis identified 14 potential bioactive compounds, with notably high expression levels in 4- and 7-year-old plants. *In vitro* antioxidant testing revealed that antioxidant activity peaked in 4-year-old plants under DPPH and FRAP assays, whereas ABTS scavenging ability was most pronounced in 6-year-old plants.

**Discussion:**

These findings elucidate the quality variations and seasonal dynamics of *A. euchroma*. Across growth years, a comprehensive evaluation identifies the 4-year growth period as the optimal harvest timing for *A. euchroma*, providing a reference for the development of standardized harvesting and quality control protocols.

## 1 Introduction

The medicinal plant Arnebia is a perennial herb belonging to the Boraginaceae family, with a long history of use in traditional Chinese medicine. Its medicinal use was first documented in Shennong’s Classic of Materia Medica (Shen Nong Ben Cao Jing) (Zhang et al., 2019). The dried roots of *Arnebia euchroma* (Royle) Johnst. and *Arnebia guttata* Bunge, both members of the Boraginaceae family, are collectively referred to as Arnebia Radix (National Pharmacopoeia Commission, 2025). According to traditional Chinese medicine, Arnebia Radix is cold in nature, sweet and salty in taste, and acts on the Heart and Liver meridians. It is known to clear heat and cool the blood, promote circulation and detoxification, induce eruptions, and eliminate macules. Clinically, it is widely used to treat febrile macules, damp-heat jaundice, purpura, burns, eczema, erysipelas, carbuncles, and ulcers (Liao and Zhang, 2020). The primary active constituents in *A.euchroma* are hydroxynaphthoquinone compounds, represented by shikonin and its derivatives. Research has demonstrated that these compounds possess a range of physiological activities, including antioxidant, anti-inflammatory, anticancer, hemostatic, immunomodulatory, antileukemic, antifertility, and hypoglycemic and hypolipidemic effects (Kaith et al., 1996; Wang et al., 2008; Dai et al., 2012; Damianakos et al., 2012; Skrzypczak et al., 2015; Cao et al., 2020; Wang et al., 2020; Ablat et al., 2021; Tan et al., 2022). The Chinese Pharmacopoeia (2025 edition) (National Pharmacopoeia Commission, 2025)designates total hydroxynaphthoquinone pigments and β,β′-Dimethylacrylalkannin as its official marker compounds. In the first edition of the Treatise on the Varieties of Chinese Medicinal Materials, published by Mr. Xie Zongwan in 1964, four botanical origins of Arnebia Radix were documented. The book also noted that *A.euchroma* was already regarded as the highest quality variant among all Arnebia Radix in the market at that time (Xie, 1963).

*A. euchroma* is primarily distributed in the temperate and subalpine regions of the western Indian Himalayas (Kumar et al., 2021). This species is distributed mainly in the Tianshan Mountains in Xinjiang. As the highest-quality source of Zicao, *A. euchroma* has become the dominant commercial variety in the herbal medicine market (Gao, 1986). According to currently available data, there are 122 documented patented traditional Chinese medicine formulations and 195 traditional Chinese medicine prescriptions that incorporate *A. euchroma* (Dai et al., 2024). In addition, *A. euchroma* is also extensively utilized in a wide range of industries, including food, cosmetics, and textile dyeing (Liao et al., 2020; Lin et al., 2023). Given its significant medicinal efficacy and market value, the market demand for *A.euchroma* has increased annually. Currently, the domestic annual demand for this medicinal material is approximately 1,200 tons. However, the supply still primarily relies on the harvesting of wild resources, with an annual output of less than 100 tons, severely constraining the development of the industry. Artificial cultivation remains the only viable strategy to meet market demand and ensure the sustainable development of the *A.euchroma* herbal industry. Seedling propagation bases have been established in several regions, including Hejing County and Zhaosu County in Xinjiang (Yang et al., 2020). However, there are still significant issues regarding the inconsistent quality of medicinal materials. Throughout the cultivation, harvesting, and processing of *A. euchroma*, it is necessary to balance key requirements, including meeting standards for indicator components, enriching active ingredients, ensuring stable efficacy, and improving yield, to promote the coordinated development of various benefits.

The bioactive constituents in medicinal plants vary significantly across different growth stages (Chen et al., 2023). Timely harvesting is not only a critical prerequisite for achieving quality standards in the Chinese herbal medicine industry but also a practical approach to ensuring high yields and superior quality (Liu et al., 2022). Investigating the relationship between the harvesting age of *A. euchroma* and its quality and yield is therefore of dual significance for enhancing both the quality of cultivated *A. euchroma* and its economic value. The accumulation pattern of bioactive compounds in plants often shows a significant time trend with the growth years (Sun et al., 2025). The optimal growth period (in years) is a critical determinant of the quality of traditional Chinese medicinal materials, with notable variations observed across plant species and growth durations. Metabolomics, a powerful tool for analyzing dynamic changes in metabolites within organisms, has been widely applied in medicinal plant research. By comprehensively deciphering the composition and content of metabolites, metabolomics can reveal the metabolic mechanisms underlying plant growth, development, and the accumulation of active components. Regarding *Codonopsis pilosula*, the content of active ingredients shows no significant difference between three-year-old and four-year-old plants; however, the active components in three-year-old *Codonopsis pilosula* remain relatively stable from September to November (Hu, 2017). In *Polygonatum odoratum*, the content of active ingredients such as polysaccharides, flavonoids, and saponins peaks in the third year of growth and then stabilizes, with no statistically significant differences observed between the fourth and sixth years and the third year (Wang et al., 2017). Numerous studies confirm that growth years significantly influence accumulation of marker compounds, secondary metabolites, and the corresponding biological activities in various medicinal plants, including *Bupleurum chinense*, *Panax ginseng* and *Achyranthes bidentata* (Tan et al., 2008; Li and Hu, 2009; Peng et al., 2025; Wang and Xiao, 2025).

It should be noted that the current Chinese Pharmacopoeia has not established specific regulations regarding the harvesting period for *A. euchroma*. This has led to a lack of unified standards for cultivation, harvesting, and quality evaluation, which may compromise the stability of the medicinal material. Therefore, determining the optimal harvesting time for this herb is important for advancing its standardization and quality control. We employed *A. euchroma* in Hejing County, Xinjiang. We measured biomass, marker compound content, and metabolite profiles at different growth stages. By integrating network pharmacology and *in vitro* antioxidant activity assays, we aim to provide scientific evidence to support standardized cultivation and harvesting of *A. euchroma*, thereby advancing its industrial development.

## 2 Materials and methods

### 2.1 Sample collection and preparation

**Figure 1:**
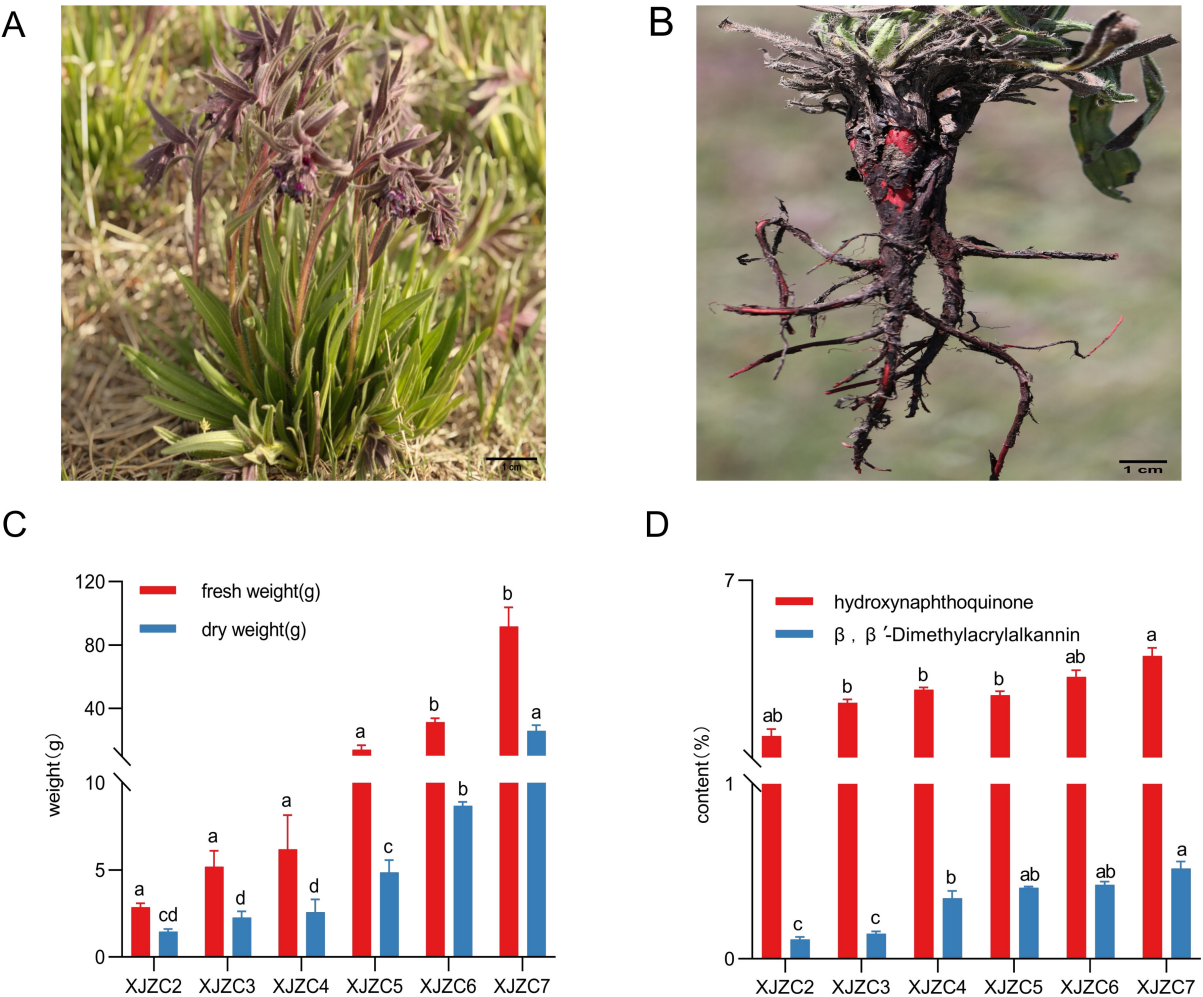
**(A)** Plant morphology of *A. euchroma*. **(B)** Root morphology of *A. euchroma*. **(C)** Root fresh weight and dry weight. **(D)** Content of hydroxynaphthoquinone and β,β’-dimethylacrylshikonin. Data are mean ± SE (n = 15); different letters indicate significant differences at *p* ≤ 0.05.

The root samples of *A. euchroma* used in this study were collected from adjacent plots under consistent management conditions at the standardized cultivation base in Hejing County, Xinjiang (latitude 42°42′33″ N, longitude 84°13′31″ E, altitude 2382.2 m). Samples included plants aged 2 to 7 years, labeled as XJZC2 to XJZC7, respectively. Researcher Guo-ping Wang, from the Institute of Traditional Chinese Medicine and Ethnic Medicine, Xinjiang Uygur Autonomous Region, identified all samples as *A.euchroma* (Royle) Johnst. For each growth year, 15 plants were selected using systematic random sampling and were randomly divided into three biological replicate groups, with five plants per group. The samples were dried at a mean daily temperature of 20–25 °C and a controlled relative humidity of 20%–30% for 15 days until a constant weight was achieved. Drying was terminated when the difference in sample mass between two consecutive measurements was less than 0.001 g, indicating that the samples had reached constant weight. Before chemical/metabolomic analysis, the five plant samples from each replicate group were combined and homogenized into a uniformly mixed powder for centralized processing and analysis. The resulting powder was then stored at -20 °C. Experimental materials are shown in **Figure 1**.

### 2.2 Determination of biomass in *A.euchroma* with different growth years

After sample collection, soil was thoroughly removed from the roots. The fresh weight was measured, and the dry weight was determined after air-drying at room temperature.

### 2.3 Determination of index components content in *A.euchroma* with different growth years

Determination of Total Hydroxynaphthoquinone Pigment Content: For the test sample, 25 mg of finely powdered *A. euchroma* was accurately weighed into a 25 mL volumetric flask, diluted to volume with ethanol, and sonicated for 1 h at room temperature. After cooling, the lost solvent was replenished. Subsequently, 3 mL of the filtrate was precisely transferred to a 10 mL volumetric flask, diluted to volume with ethanol, and mixed to obtain the test solution. Using a UV spectrophotometer with ethanol as the blank, the absorbance was measured at 516 nm following a wavelength scan from 400 nm to 600 nm.

Determination of β,β′-Dimethylacrylalkannin Content: Approximately 200 mg of *A.euchroma* powder was accurately weighed and transferred into a conical flask. 10 mL of petroleum ether (60-90 °C) was added, and the initial total mass was recorded. The mixture was sonicated (power: 250 W, frequency: 33 kHz) for 30 minutes for extraction. After cooling to room temperature, the total mass was recorded again, and the mass loss was compensated by adding additional petroleum ether. The mixture was thoroughly mixed and filtered. An aliquot of 5 ml filtrate was collected in a new conical flask and evaporated to dryness. The residue was dissolved in an appropriate amount of acetonitrile, vortexed for 30 seconds, and then transferred to a 10 mL volumetric flask. The solution was diluted to volume with acetonitrile, mixed well, and filtered through a 0.45 μm microporous membrane. The resulting filtrate was collected as the final test solution. Chromatographic conditions: ACE EXCEL 5 SUPER C18 column (4.6 mm × 250 mm); mobile phase: acetonitrile (C) - 0.05% formic acid aqueous solution (D) with gradient elution (0–5 min, 66%–70% C; 5–20 min, 70%–80% C; 20–25 min, 80%–85% C; 25–38 min, 85%–85% C; 38–45 min, 85%–66% C); flow rate: 0.8 mL·min^−^¹; column temperature: 30°C; detection wavelength: 275 nm; injection volume: 10 μL.

### 2.4 Non-targeted metabolomics analysis of *A.euchroma* with different growth years

The fresh plants were desiccated and ground into powder. The powder (50 mg ± 2 mg) were taken and lyophilized, mixed with beads and 500 μL of extraction solution (MeOH: ACN:H_2_O, 2:2:1 (v/v/v)) which contain deuterated internal standards. The mixed solutions were vortexed for 30 s, homogenized (35 Hz, 240 s), sonicated for 5 min in 4°C water bath. The homogenization and sonication repeated 3 times. Then the mixed solution incubated for 30 min at -40°C to precipitate proteins. Then the samples were centrifuged at 12000 rpm for 15 min at 4°C. The supernatant liquids were transferred to fresh vials to incubate for 10 min and they were centrifuged at 12000 rpm for 15 min at 4°C again. The new supernatant liquids were transferred to fresh glass vials for analysis. The quality control (QC)sample was prepared by mixing an equal aliquot of the supernatant of samples.LC-MS/MS analyses were performed using an UHPLC system (Vanquish, Thermo Fisher Scientific) with a Phenomenex Kinetex C18 (2.1 mm × 100 mm, 2.6 μm) coupled to Orbitrap Exploris 120 mass spectrometer (Orbitrap MS, Thermo). The mobile phase A:0.01% acetic acid in water; mobile phase B:IPA: ACN (1:1,v/v). The auto-sampler temperature was 4°C, and the injection volume was 2 μL. The Orbitrap Exploris 120 mass spectrometer was used for its ability to acquire MS/MS spectra on information-dependent acquisition (IDA) mode in the control of the acquisition software (Xcalibur, Thermo).

### 2.5 Network pharmacology research

The CAS numbers of the differentially expressed metabolites initially screened by non-targeted metabolomics were entered into the Traditional Chinese Medicine Systems Pharmacology Database and Analysis Platform (TCMSP). Potential active compounds were filtered with the criteria of oral bioavailability (OB) ≥30% and drug-likeness (DL) ≥0.18. The SMILES strings of these active compounds from *A. euchroma* were then submitted to SwissTargetPrediction (http://www.swisstargetprediction.ch) with the species set to “Homo sapiens” for target prediction. The exported target prediction results were filtered by a probability threshold of ≥0.1. Finally, the active compounds and their predicted targets were imported into Cytoscape 3.8.2 to construct an Active Compound-Target Network.

### 2.6 Antioxidant assays

The scavenging capacities against ABTS and DPPH radicals were determined using specific commercial assay kits (Solarbio, China). The half-maximal inhibitory concentration (IC_50_) was calculated, where a lower IC_50_ value indicates a stronger scavenging ability. Additionally, the total antioxidant capacity (T-AOC) was assessed using the corresponding kit (Solarbio, China) based on the Ferric Reducing Antioxidant Power (FRAP) method.

### 2.7 Data statistics and analysis

The raw metabolomics data were converted into mzXML format using ProteoWizard software, followed by metabolite identification using a custom-developed R package against the Biotree TCM (V1.0) and BT-HERB (V1.0) databases. Visualization analysis was performed using another in-house R package. Principal Component Analysis (PCA) and Orthogonal Partial Least Squares-Discriminant Analysis (OPLS-DA) were conducted using SIMCA 14.1 software. Differential metabolites were selected based on the Variable Importance in Projection (VIP) values derived from the OPLS-DA model and t-test results, with thresholds set at VIP >1 and *P* < 0.05. Statistical analyses were performed using SPSS version 20.00. The Brown-Forsythe analysis of variance test was applied to determine the overall significance among treatment means when the assumption of homogeneity of variances was violated. For *post hoc* pairwise comparisons, Dunnett’s T3 test was used at a significance level of *p* ≤ 0.05. Visualizations were generated using R 4.4.3, Excel, and GraphPad Prism.

## 3 Results

### 3.1 Biomass changes in *A. euchroma* at different growth years

The results revealed significant differences in *A. euchroma* biomass across different growth years (*P* < 0.05). As growth years increased, both the fresh and dry weights of *A. euchroma* roots showed a rising trend. Fresh weight increased steadily from XJZC2 to XJZC4, followed by a marked rise beginning at XJZC5. Similarly, dry weight grew steadily from XJZC2 to XJZC5, with a significant surge observed from XJZC6 onward (**Figure 1C**).

### 3.2 Content of characteristic components in *A. euchroma* at different growth years

We observed significant variations in the contents of hydroxynaphthoquinone pigments and β,β′-dimethylacrylalkannin in *A. euchroma* over different harvesting years (*P* < 0.05). The total hydroxynaphthoquinone pigment content followed a dynamic pattern: an initial increase, followed by a decline, and then a resurgence. The lowest content (1.73%) occurred in XJZC2, while the highest (4.44%) was measured in XJZC7. The concentrations decreased in the following order: XJZC7 > XJZC6 > XJZC4 > XJZC5 > XJZC3 > XJZC2. In all samples from XJZC2 to XJZC7, total hydroxynaphthoquinone pigment levels exceeded the Pharmacopoeia standard (≥ 0.80%) (**Figure 1C**). The content of β,β′-dimethylacrylalkannin increased with harvesting year. No significant difference was observed between XJZC2 and XJZC3, but levels began rising steadily from XJZC4. The lowest content (0.11%) was recorded in XJZC2, and the highest (0.52%) in XJZC7. Notably, the contents in XJZC2 and XJZC3 did not meet the Pharmacopoeia standard (≥ 0.30%) (**Figure 1D**).

### 3.3 Analysis of co-expressed metabolites in *A. euchroma* across different growth years

**Figure 2:**
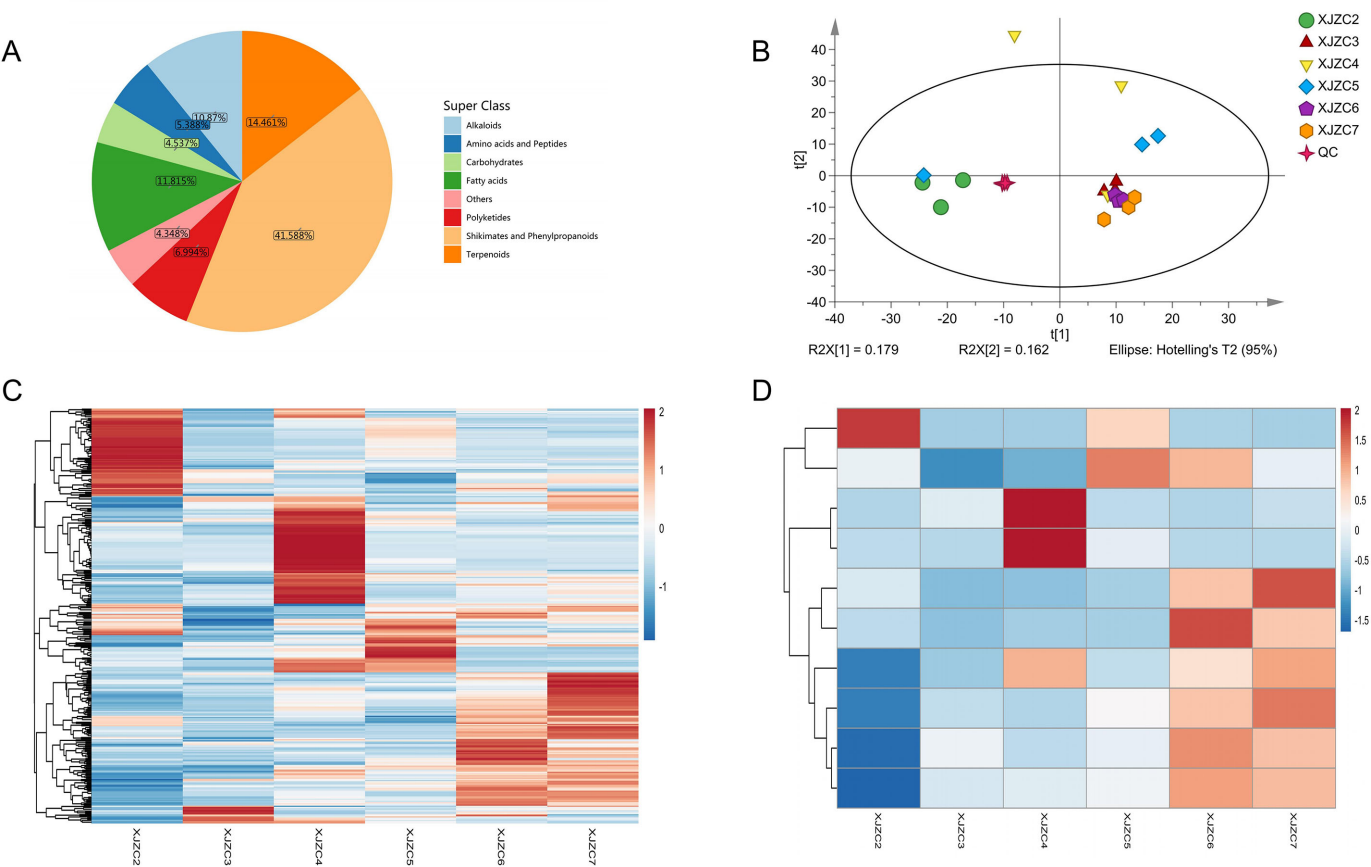
**(A)** Proportion of metabolites in *A. euchroma*. **(B)** PCA score plot of *A. euchroma* at different growth years. **(C)** Clustered heatmap of differential metabolites in *A. euchroma* across different growth years. **(D)** Clustered heatmap of the top 10 significantly differentially expressed metabolites.

As shown in (**Supplementary Figures S1A, B**), the total ion chromatograms (TICs) of all QC samples showed substantially overlapping peak response intensities and retention times, indicating excellent instrument precision. The multivariate control chart (**Supplementary Figure S1C**) demonstrated that both QC and experimental samples fluctuated within the range of positive and negative standard deviations, confirming that instrumental variation remained within acceptable limits and showed adequate stability. We identified a total of 1,058 metabolites in *A.euchroma* across different growth years, including 575 in positive ion mode and 483 in negative ion mode. These metabolites comprised 154 terpenoids, 131 flavonoids, 125 fatty acids, 115 alkaloids, 74 polyketides, 66 lignans, 59 coumarins, 57 amino acids and derivatives, 54 phenylpropanoids, 48 aromatic compounds, 48 carbohydrates, 46 phenolic acids, and 81 additional compounds (**Figure 2A**). Among these, terpenoids, flavonoids, fatty acids, and alkaloids were the most abundant compound classes in *A. euchroma*. PCA (**Figure 2B**) revealed that QC samples clustered tightly and were clearly separated from the experimental sample groups, confirming strong and excellent QC repeatability. These results underscore the reliability of the experimental methods described above. The sample groups were mutually separated, with the XJZC2 group showing the most pronounced separation from the others.

OPLS-DA results showed that samples from each group were well separated within the confidence interval, demonstrating strong and good discrimination efficiency. Samples from different groups were clearly distinct and mutually separated, while those within the same group clustered closely, indicating substantial metabolic differences among *A. euchroma* from different years. For the permutation random models (**Supplementary Figures S2B, D**, and **S3B**), the predictive ability (Q²) was consistently lower than that of the original models, supporting their robustness. In some cases, the Q² values of permutation random models (**Supplementary Figures S2E, D**) were not lower than those of the original models; both the R²Ymodel and their explainable rates for dependent variables (R²Y) approached 1. These results indicate that the models exhibited strong correlations near 1, suggesting extremely strong interpretability and high predictive power. In conclusion, this dataset demonstrates excellent stability and reliability; the models are statistically valid and meaningful, without signs of overfitting, and differential metabolites can be reliably identified by further screening based on the variable importance in the projection (VIP) index.

### 3.4 Analysis of differential metabolites in *A. euchroma* across different growth years

Based on a significance threshold of *P* < 0.05, we identified a total of 355 significantly differential metabolites in *A. euchroma* across the six growth years. These included 44 terpenoids, 35 fatty acids, 34 flavonoids, 31 carbohydrates, 26 amino acids and derivatives, 21 lignans, 18 alkaloids, 16 polyketides, 15 phenylpropanoids, 14 coumarins, 11 phenolic acids, 10 aromatic compounds, and 22 other compounds. Clustering heatmap analysis (**Figure 2C**) further verified the distribution and expression levels of differential metabolites across various growth years of *A.euchroma.* The ten most significantly differential metabolites were Cedrin, 1-(3,4-dihydroxyphenyl)-6,7-dihydroxy-1,2-dihydronaphthalene-2,3-dicarboxylic acid, Schizotenuin A, Lotaustralin, Kuwanon C, (R)-naringenin, Tanegoside, Oreganol, Toxol, and Hinokiflavone. The heatmap visually depicts the expression levels of the top 10 differential metabolites across different growth years of *A. euchroma* (**Figure 2D**).

**Figure 3:**
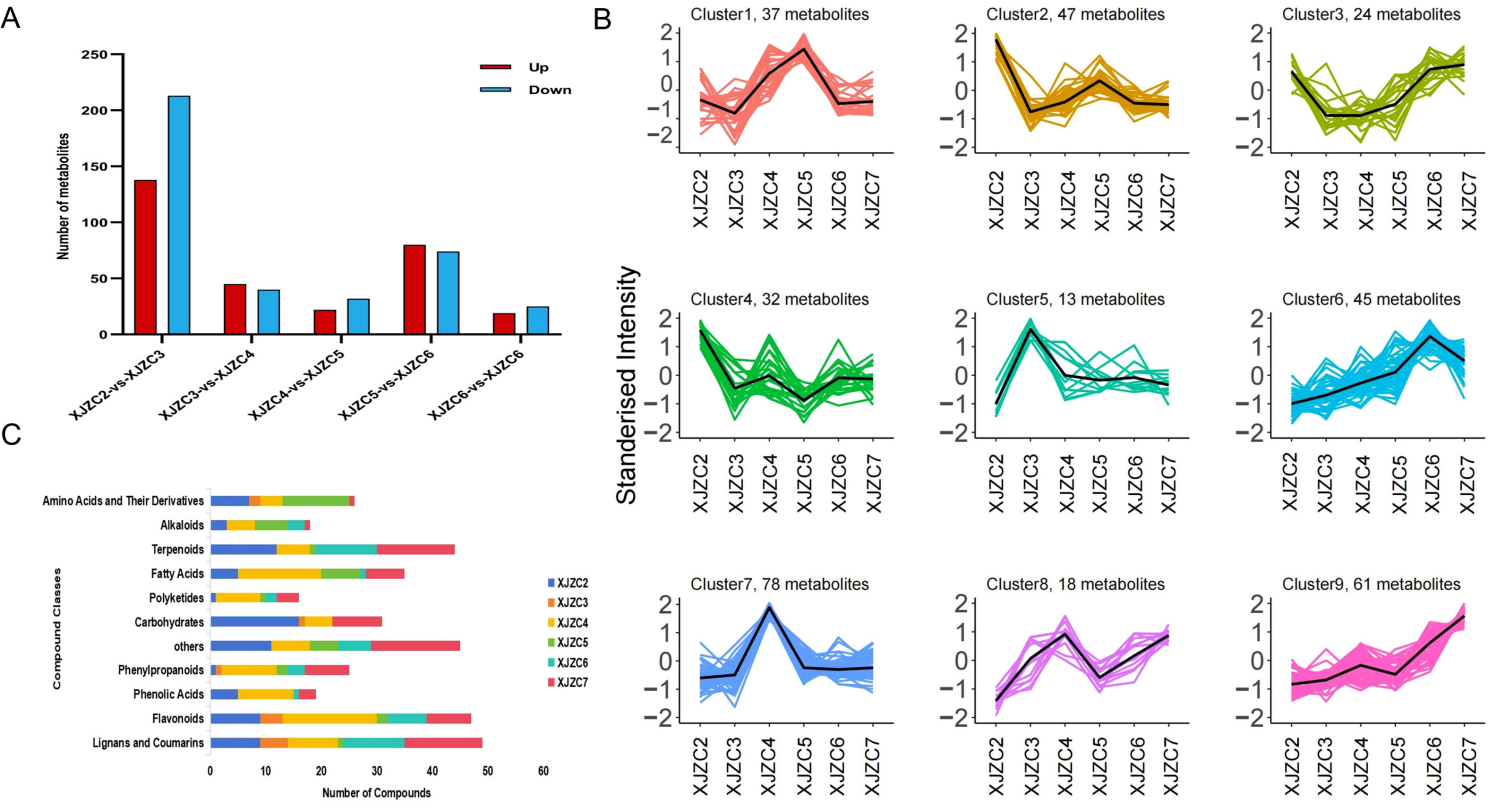
**(A)** Statistical distribution of up-regulated, down-regulated, and total differential metabolites in *A. euchroma* at different growth years. **(B)** K-Means clustering analysis of differential metabolites. **(C)** Classification of metabolites with distinct accumulation patterns.

Based on the criteria of *P* < 0.05 and VIP > 1, we identified 351 differential metabolites in the XJZC2-vs-XJZC3 group, including 138 significantly upregulated and 213 significantly downregulated compounds. In the XJZC3-vs-XJZC4 group, we detected 88 differential metabolites, including 45 that were significantly upregulated, 40 that were significantly downregulated, and 3 that showed no significant change. The XJZC4-vs-XJZC5 group yielded 54 differential metabolites, with 22 significantly upregulated and 32 significantly downregulated. In the XJZC5-vs-XJZC6 group, we identified 154 differential metabolites, including 80 significantly upregulated and 74 significantly downregulated. The XJZC6-vs-XJZC7 group showed 44 differential metabolites, with 15 significantly upregulated and 29 significantly downregulated. According to the up- and downregulation patterns of differential metabolites (**Figure 3A**), *A. euchroma* showed the most pronounced metabolic changes, with the greatest change between the second and third years. The most significantly affected metabolite classes included terpenoids, flavonoids, fatty acids, amino acids, lignans, and coumarins. Most flavonoids, lignans, and coumarins were upregulated, whereas most terpenoids, fatty acids, and amino acids were downregulated. From the fourth year onward, the up/downregulation trends of metabolites remained relatively stable.

K-means clustering analysis was performed on all differential metabolites (**Figure 3B**), and these metabolites were categorized into nine distinct clusters based on their accumulation patterns. Notably, 96 metabolites (Clusters 7 and 8) showed higher levels in XJZC4, 85 metabolites (Clusters 3 and 9) in XJZC7, 79 metabolites (Clusters 2 and 4) in XJZC2, 45 metabolites (Cluster 6) in XJZC6, 37 metabolites (Cluster 1) in XJZC5, and 13 metabolites (Cluster 5) in XJZC3. Further classification of these six major clustering patterns (**Figure 3C**) revealed distributions of compounds. Flavonoids, phenolic acids, polyketides, phenylpropanoids, and fatty acids were most abundant in XJZC4. Most Lignans, coumarins, and terpenoids accumulated primarily in XJZC7, while alkaloids, amino acids, and their derivatives were more concentrated in XJZC5. Most carbohydrates were enriched in XJZC2. These findings indicate that the changing trends in metabolite accumulation in *A. euchroma* follow distinct, growth-year–dependent patterns, exhibiting regularity.

### 3.5 Kyoto encyclopedia of genes and genomes pathway enrichment analysis of differential metabolites in *A. euchroma* with different growth years

Based Metabolic pathways of metabolites were annotated based on the KEGG database, and significantly altered metabolic pathways were analyzed to explore the regularity of substance variations during its growth and development. KEGG pathway enrichment analysis was performed, with *P* ≤ 0.05 set as the criterion for defining significant pathway enrichment. The pathway enrichment results were presented as a scatter plot. Using the XJZC2 Group as a control, pathway enrichment analysis was conducted on the screened differential metabolites, and The XJZC2-vs-XJZC3 group was enriched in Purine metabolism, Biosynthesis of unsaturated fatty acids, Starch and sucrose metabolism, and Riboflavin metabolism. The XJZC2-vs-XJZC4 group was enriched in Galactose metabolism, Starch and sucrose metabolism, Purine metabolism, Cysteine and methionine metabolism, Pyrimidine metabolism, Neomycin, kanamycin and gentamicin biosynthesis, Tyrosine metabolism, Citrate cycle (TCA cycle), and Fructose and mannose metabolism. The XJZC2-vs-XJZC5 group was enriched in Valine, leucine and isoleucine biosynthesis, Alanine, aspartate and glutamate metabolism, Arginine biosynthesis, Starch and sucrose metabolism, Nitrogen metabolism, Valine, leucine and isoleucine degradation, Tyrosine metabolism, Galactose metabolism, and Glycine, serine and threonine metabolism. The XJZC2-vs-XJZC6 group was enriched in Starch and sucrose metabolism, Galactose metabolism, Glycine, serine and threonine metabolism, Purine metabolism, Valine, leucine and isoleucine biosynthesis, Tyrosine metabolism, Cysteine and methionine metabolism, Citrate cycle (TCA cycle), Pyrimidine metabolism, Pyruvate metabolism, and Alanine, aspartate and glutamate metabolism. The XJZC2-vs-XJZC7 group was enriched in Starch and sucrose metabolism, Purine metabolism, Galactose metabolism, Biosynthesis of unsaturated fatty acids, Tyrosine metabolism, Citrate cycle (TCA cycle), Valine, leucine and isoleucine biosynthesis, Phenylalanine metabolism, and Caffeine metabolism. These metabolic pathways may play roles in the growth, development, and substance accumulation of *A. euchroma*, leading to differences in metabolites across different growth years (**Supplementary Figure S4**).

### 3.6 Network pharmacology analysis

To explore the potential active components of *A. euchroma*, the TCMSP database platform was used to screen the potential active components of the 355 differential metabolites obtained from the above screening, and a total of 151 differential metabolites were detected. Using oral bioavailability (OB) ≥ 30% and drug-likeness (DL) ≥ 0.18 as the criteria, 14 potential active components were screened out, as shown in **Table 1**. There were 7 flavonoids, 2 lignans, and 1 each of terpenoids, polyketides, nucleosides, coumarins, and alkaloids. The results indicate that flavonoids may represent the main potential active components of *A. euchroma*.

**Table 1:** Potential active components of *A. euchroma.*

Molecule ID	CAS number	TCMSP OB DL	Potential active component	Classification
MOL006967	146-80-5	44.72 0.21	Xanthosine	nucleosides
MOL007245	1592-70-7	60.16 0.26	Kaempferol 3-methyl ether	flavonoids
MOL000098	117-39-5	46.43 0.28	Quercetin	flavonoids
MOL001040	93602-28-9	42.36 0.21	Naringenin	flavonoids
MOL003095	18103-41-8	51.96 0.41	Corymbosin	flavonoids
MOL003378	6468-55-9	33.94 0.43	Demethylwedelolactone	flavonoids
MOL005259	479-90-3	49.55 0.48	Artemetin	flavonoids
MOL010565	1260-17-9	35.61 0.84	Carminic_acid	polyketides
MOL003196	74285-86-2	48.5 0.44	Triptophenolide	terpenoids
MOL009047	526-06-7	33.29 0.62	Eudesmin	lignans
MOL000533	33464-71-0	40.19 0.81	Tracheloside	lignans
MOL009330	128-62-1	53.29 0.88	narcotine	alkaloids
MOL013083	93-39-0	38.35 0.32	skimmin	coumarins
MOL001798	13241-33-3	71.17 0.27	Hesperetin-7-O-neohesperidoside	flavonoids

**Figure 4:**
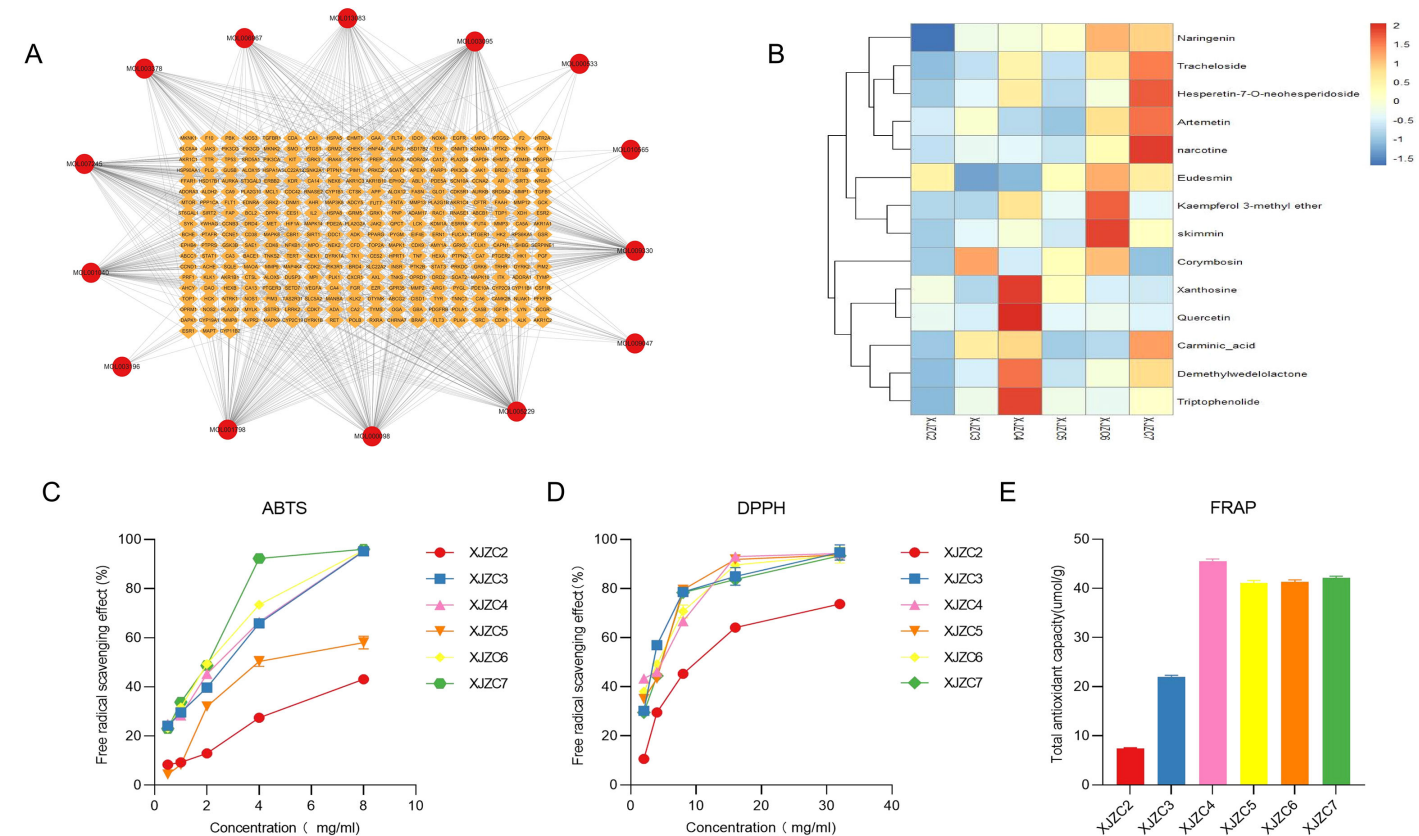
**(A)** Potential active ingredient-potential target network of *A. euchroma*; the red circle represents potential active ingredients, and the yellow diamond represents potential target genes. **(B)** Clustering heatmap of potential bioactive components in *A. euchroma* across different growth years. **(C)** ABTS radical scavenging activity. **(D)** DPPH radical scavenging activity. **(E)** Ferric reducing antioxidant power (FRAP).

To investigate the potential protein targets of the active components in *A.euchroma*, the target information of the 14 candidate active compounds was predicted using SwissTargetPrediction. With a screening threshold of Probability ≥ 0.1 and after deduplication, 328 potential targets were identified. These findings suggest that the active components of *A.euchroma* may act on a diverse range of targets. However, current research on the interactions between these screened compounds and their potential targets remains limited, warranting further experimental validation in future studies. To visualize the potential relationships between the active components and their predicted targets, the data were imported into Cytoscape 3.10.1, generating a Compound-Target interaction network (**Figure 4A**). Additionally, the 14 candidate active components identified via network pharmacology were subjected to cluster heatmap analysis to assess their expression levels in *A. euchroma* of different growth years. The results revealed significant variations in the abundance of these compounds across samples, with most exhibiting higher expression in XJZC4 and XJZC7 (**Figure 4B**).

### 3.7 *Vitro* antioxidant activity of *A. euchroma* across different growth years

As determined by the ABTS method (**Figure 4C**), the IC_50_ values of *A. euchroma* samples across different growth years against ABTS radicals ranged from 1.635 to 12.237 mg/mL. The 7-year-old *A. euchroma* exhibited the strongest ABTS radical scavenging capacity with an IC_50_ of 1.635 mg/mL, whereas the 2-year-old sample showed the weakest activity (IC_50_ = 12.237 mg/mL).

Using the DPPH method (**Figure 4D**), the IC_50_ values for DPPH radicals ranged from 3.497 to 10.264 mg/mL. The 4-year-old *A. euchroma* demonstrated the highest DPPH scavenging ability (IC_50_ = 3.497 mg/mL), while the 2-year-old sample again showed the lowest activity (IC_50_ = 10.264 mg/mL).

The FRAP assay results (**Figure 4E**) revealed FRAP values ranging from 7.473 to 45.565 μmol/g. The 4-year-old sample displayed the strongest antioxidant activity (45.565 μmol/g), consistent with the trend observed in the DPPH assay, while the 2-year-old sample had the lowest FRAP value (7.473 μmol/g).

## 4 Discussion

“Timely harvesting makes herbs effective medicine, while overdue harvesting turns them into ordinary grass”. Harvest timing plays a critical role in both the quality and yield of medicinal herbs, making it one of the most important stages in the production process (Huang et al., 2024). The accumulation of active compounds and biomass serves as a key indicator for determining the optimal harvest period and number of growth years for perennial medicinal plants (Tian, 2018). Harvesting at the appropriate time maximizes both the medicinal value and economic return of these herbs. For *A. euchroma*, the timing and duration of harvest are critical under artificial cultivation. Harvesting too early results in low biomass and insufficient levels of active compounds, while harvesting too late increases labor and time costs.

Biomass accumulation is a critical indicator of crop growth and development. In Particular, allocating a higher proportion of dry matter to harvested plant parts is a prerequisite for achieving both superior quality and high yield (Yue et al., 2024). A study by Zhang et al. (2021) on *Fagopyrum dibotrys* demonstrated that although biomass increases with extended growth duration, the optimal harvesting period is two years, as concurrent declines in economic benefits (including output value and cost-benefit ratio) occur. This ensures sustainable profits for medicinal herb growers. We assessed both biomass and marker compound levels in *A. euchroma* across different growth years and found that biomass increased with plant age. Considering the balance between cost and economic returns, the fourth year is recommended as the optimal harvesting time.

The intrinsic chemical profile is fundamental to the quality of Traditional Chinese Medicine (TCM) and serves as the material basis for its therapeutic effects (Liu et al., 2025). Marker components, which are not only key indicators for quality assessment in the Chinese Pharmacopoeia but also often represent the primary bioactive constituents of medicinal materials (Liu, 2016; Liu, 2017; National Pharmacopoeia Commission, 2025), were quantified Analysis of *A. euchroma* across different growth years revealed a gradual increase in the content of these marker components with prolonged growth duration, indicating that growth age is a critical factors in determining the quality of medicinal herbs. Metabolomics has become a pivotal technology for establishing quality evaluation systems for Chinese herbal medicines. Previous studies have used metabolomic approaches to elucidate the dynamic changes in active components of medicinal materials such as ginseng, providing a scientific basis for determining optimal harvesting times (Fiehn, 2002; Li et al., 2023; Yang et al., 2023; Zhou et al., 2024). The present study revealed that the highest diversity and relatively peak concentrations of flavonoids, phenolic acids, naphthoquinones, and other bioactive compounds were observed in 4-year-old *A. euchroma*. These compounds constitute the material basis for this herb’s medicinal efficacy, serving as core components responsible for its documented biological activities including antitumor, anticancer, antimicrobial, anti-fertility, and antioxidant effects (Tao et al., 2025). The observed trend in variation of flavonoid components aligns with the mid-growth-phase accumulation pattern of secondary metabolites reported in various perennial medicinal plants (Wei et al., 2023; Kuang et al., 2020; Li et al., 2024; Bo et al., 2020). For instance, flavonoids in Dendrobium officinale and Scutellaria baicalensis reach their peak levels at 3–5 years of growth (Wei et al., 2023; Li et al., 2024). This accumulation peak is considered to be associated with the transition between plant developmental stages: during the mid-phase, as vegetative growth approaches maturity, the resource allocation strategy shifts from biomass increase toward the synthesis of defensive secondary metabolites (Kuang et al., 2020). Notably, not all flavonoids peaked in the fourth year, suggesting that there may be fine-tuned branch-specific regulation within their biosynthetic network. Activation of common upstream pathways likely drives the synchronous accumulation of the main flavonoid pool. In contrast, downstream-specific modification steps—such as glycosylation and acylation—appear to be independently regulated, leading to distinct accumulation dynamics for certain flavonoids (Wei et al., 2023). This complex accumulation pattern closely resembles the reported divergence in accumulation trends between glycosides and aglycones in Scutellaria baicalensis (Wei et al., 2023).

Untargeted metabolomics analysis revealed that flavonoids and phenolic acids accumulated most prominently in the roots of 4-year-old *A. euchroma*. This metabolic profile was supported by KEGG pathway enrichment analysis, which indicated an upregulation of primary metabolic pathways (such as carbohydrate metabolism and the tricarboxylic acid cycle) and tyrosine metabolism. These coordinated changes likely supplied the necessary precursors and energy for the biosynthesis of these phenylpropanoid compounds. *In vitro* antioxidant assays further corroborated these findings, as the 4-year-old samples exhibited the strongest radical-scavenging capacity in both the DPPH and FRAP tests, consistent with the high flavonoid abundance during this growth stage.

Network pharmacology has emerged as a robust methodology for scientifically elucidating the bioactivities of Chinese herbal medicines (Lee et al., 2023). Network pharmacology analysis identified 14 potential bioactive components—including quercetin, demethylwedelolactone, artemetin, and naringenin—whose mechanisms of action are primarily associated with key cancer-related targets such as CDK2 and GSK3B (Chen et al., 2019; Markowitsch et al., 2021). Multiple studies have demonstrated that these potential bioactive components possess a spectrum of pharmacological activities, including antioxidant, anti-inflammatory, and anticancer effects (Di Petrillo et al., 2022; Zhu et al., 2022). Thus, the network pharmacology results provided supportive validation for the bioactive components highlighted by the metabolomics data. As the growth period extended to 7 years, a shift in the metabolic profile was observed. KEGG pathway analysis showed increased activity in processes such as unsaturated fatty acid biosynthesis. Although several active components identified earlier remained detectable, the *in vitro* antioxidant activity profile changed, with the ABTS radical scavenging capacity reaching its highest level. This suggests that the plant’s physiological state and its corresponding antioxidant strategy may vary across different growth years.

In summary, this study thoroughly investigated the intrinsic quality formation patterns of *A. euchroma* across different growth years through a comprehensive analysis of biomass accumulation, marker component content, metabolite dynamics, and *in vitro* antioxidant activity. By integrating multiple factors including biomass growth marginal utility, cost-benefit ratio, bioactive constituents, and *in vitro* biological activity, the optimal harvesting period for *A. euchroma* was determined to be four years. These findings provide crucial scientific evidence to guide the standardized cultivation and rational harvesting of this valuable medicinal resource.

## 5 Conclusions

This study elucidates the accumulation patterns of biomass, marker components, and metabolites in *A. euchroma* across different growth years. The results demonstrate that 4-year-old plants achieve an optimal balance between medicinal efficacy and economic viability. Both network pharmacology analysis and *in vitro* antioxidant assays consistently confirmed the superior pharmacological value of the 4-year harvested material. These results underline the importance of specifying harvesting periods and plant parts to ensure consistent quality in *A. euchroma*. The insights would contribute to the development of standardized harvesting protocols and quality control measures for *A. euchroma*. Based on the above conclusions, future research will further determine the optimal harvesting month for *A. euchroma* and integrate multi-omics technologies to deeply elucidate the biosynthetic pathways and regulatory mechanisms of key active ingredients such as shikonin. Subsequently, this could serve as a foundation for exploring the development of more refined quality evaluation protocols, thereby supporting the sustainable utilization of *A. euchroma* resources and the steady development of the industry.

## Data Availability

The original contributions presented in the study are included in the article/**Supplementary Material**. Further inquiries can be directed to the corresponding authors.
