# Supplementary material for: Quality evaluation of Arnebia euchroma with different growth years based on metabolomics and antioxidant activity
Source: Front Plant Sci. 2026 Jan 29;17:1735489. doi: 10.3389/fpls.2026.1735489 (PMC12894223; doi:10.3389/fpls.2026.1735489)
Supplement: Supplementary file 3 [file DataSheet3.docx]

Supplementary Material


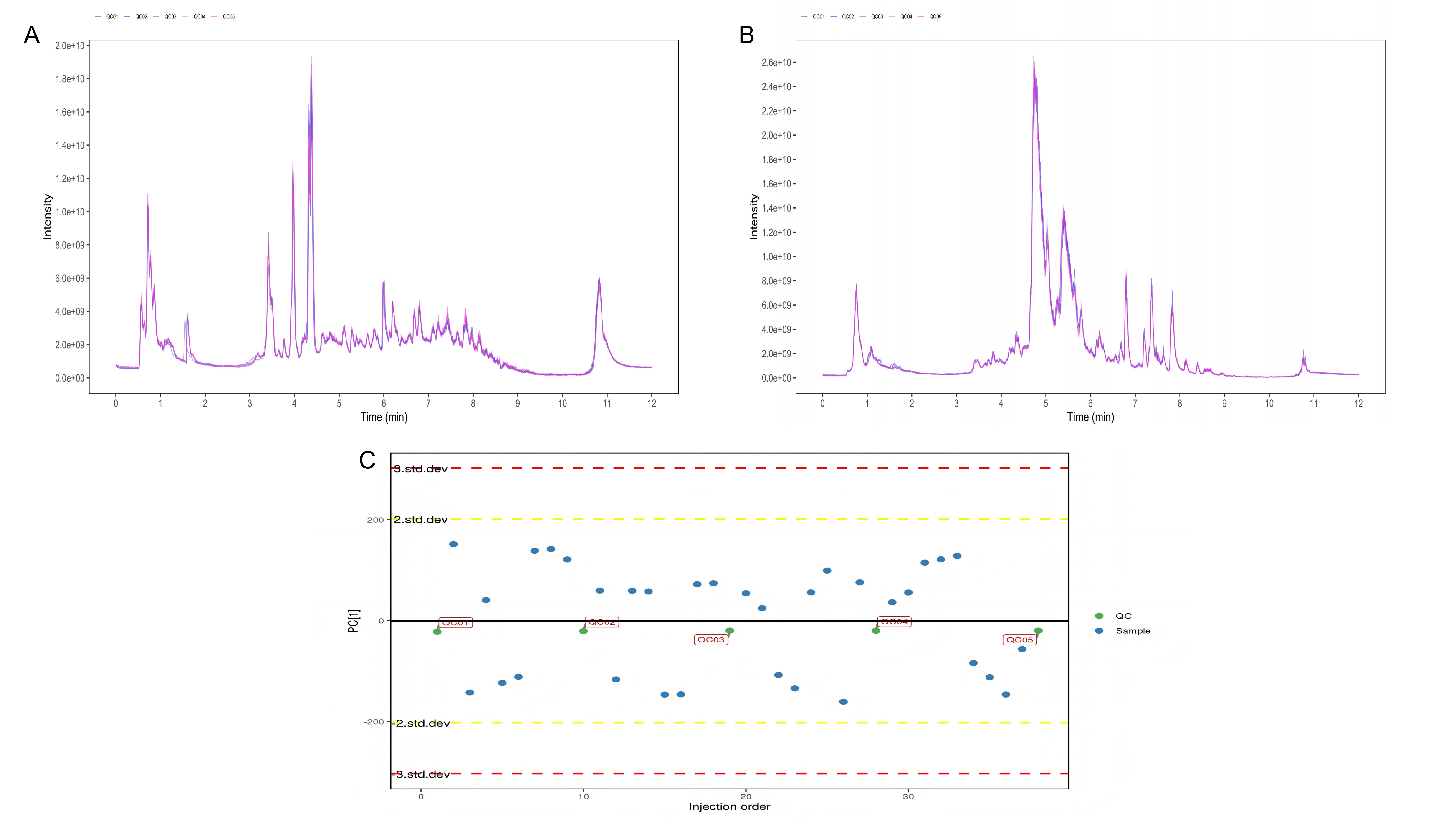


**Supplementary Figure 1.** shows the quality control analysis of metabolites detected in A.euchroma. A and B: Total ion chromatograms of QC samples in positive and negative modes. C: Multivariate control chart.


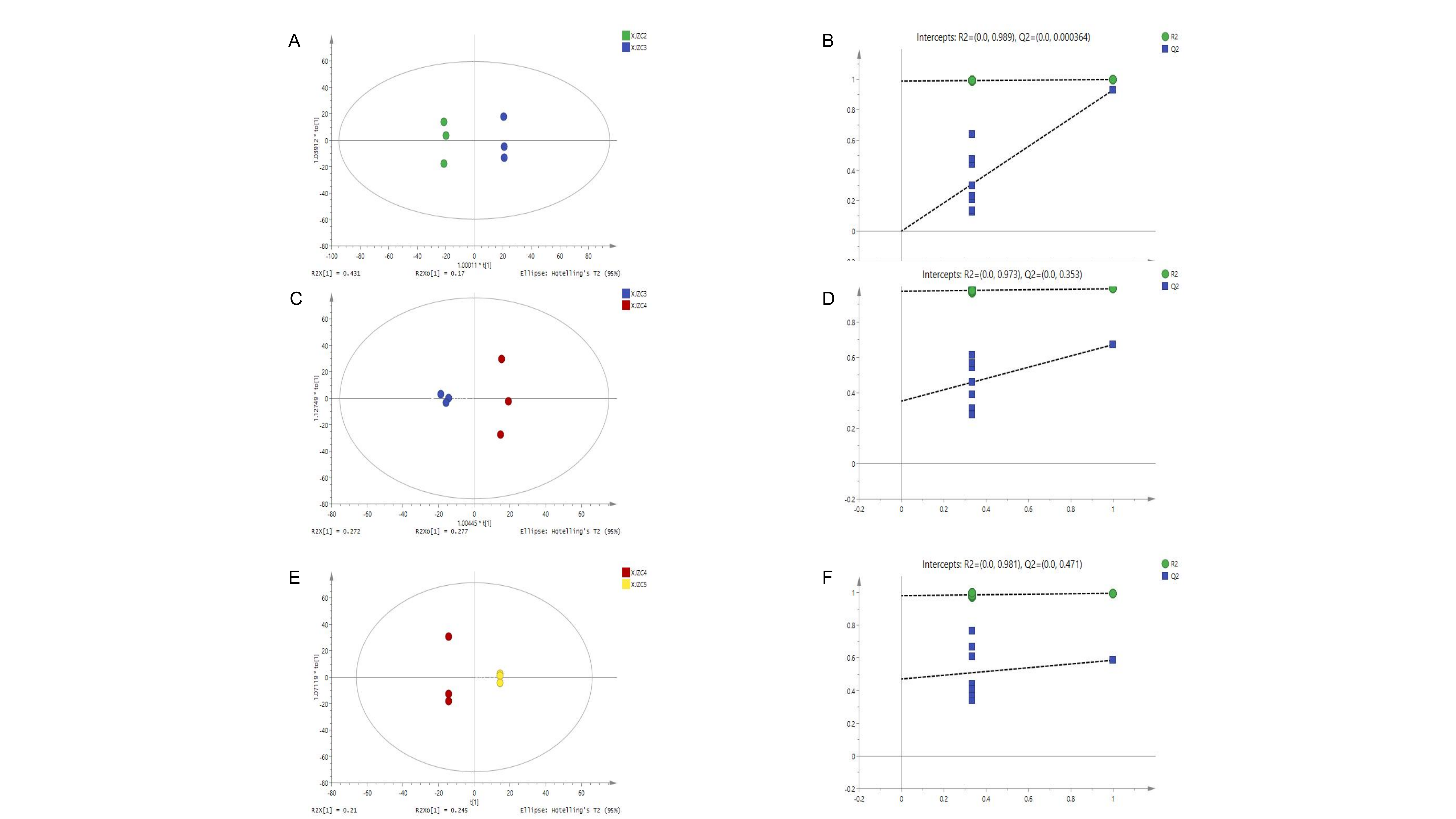


**Supplementary Figure 2.** the OPLS-DA plots and permutation test plots of metabolites across different growth years. A: OPLS-DA plot for XJZC2-vs-XJZC3 group. B: Permutation test plot for XJZC2-vs-XJZC3 group. C: OPLS-DA plot for XJZC3-vs-XJZC4 group. D: Permutation test plot for XJZC3-vs-XJZC4 group. E: OPLS-DA plot for XJZC4-vs-XJZC5 group. F: Permutation test plot for XJZC4-vs-XJZC5 group.


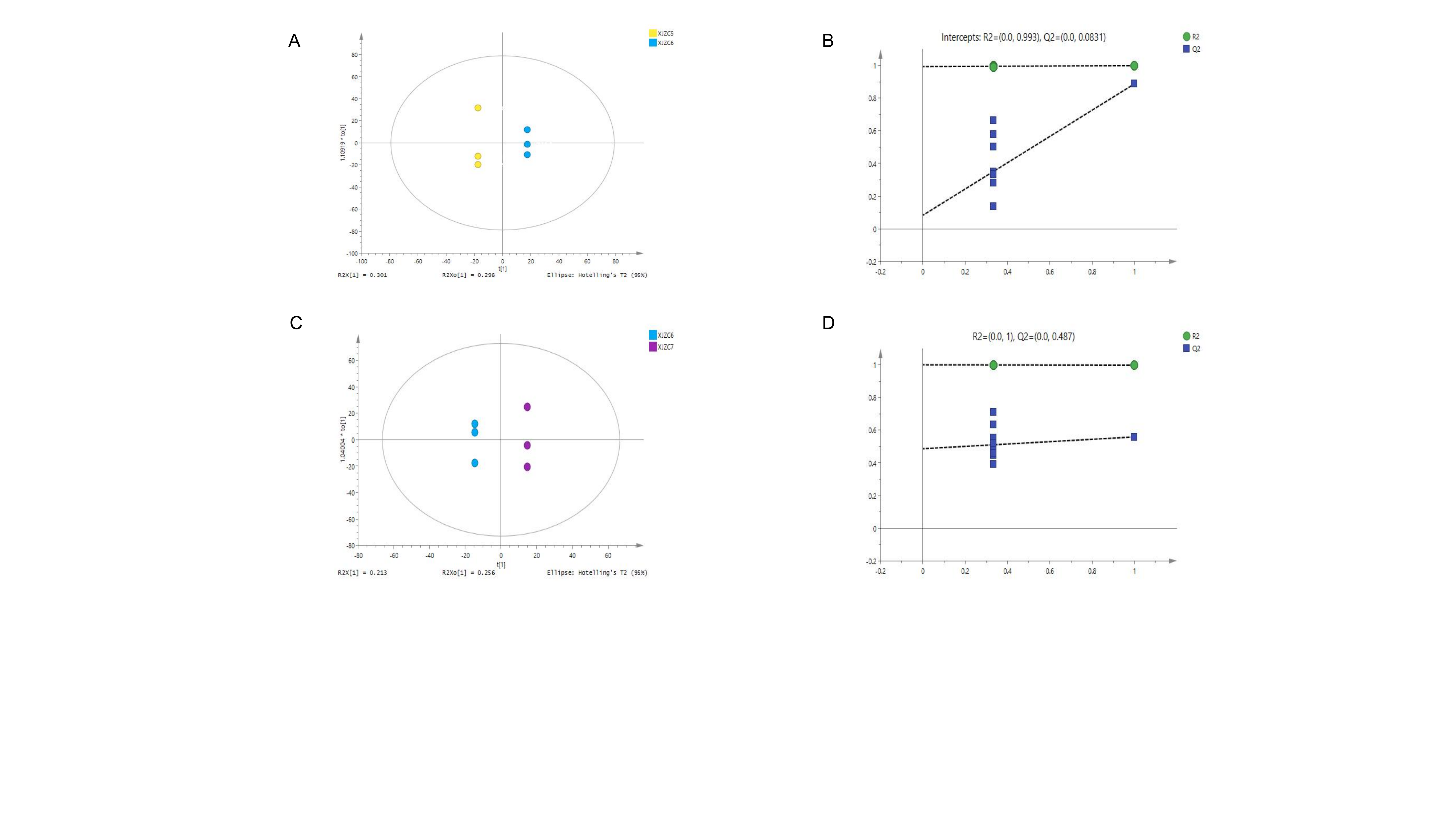


**Supplementary Figure 3.** .the OPLS-DA plots and permutation test plots of metabolites across different growth years. A: OPLS-DA plot for XJZC5-vs-XJZC6 group. B: Permutation test plot for XJZC5-vs-XJZC6 group. C: OPLS-DA plot for XJZC6-vs-XJZC7 group. D: Permutation test plot for XJZC6-vs-XJZC7 group.





**Supplementary Figure 4.**shows the enrichment analysis plots of differential metabolites across different growth years. A: Enrichment analysis plot for XJZC2-vs-XJZC3 group. B: Enrichment analysis plot for XJZC2-vs-XJZC4 group. C: Enrichment analysis plot for XJZC2-vs-XJZC5 group. D: Enrichment analysis plot for XJZC2-vs-XJZC6 group. D: Enrichment analysis plot for XJZC2-vs-XJZC7 group.
